# Thermally responsive AIE-active polyurethanes based on a tetraaniline derivative†

**DOI:** 10.1039/d0ra06193j

**Published:** 2020-11-13

**Authors:** Beibei Liu, Kun Wang, Hao Lu, Mingming Huang, Zhigang Shen, Jiping Yang

**Affiliations:** Beijing Key Laboratory for Powder Technology Research & Development, School of Aeronautical Science and Engineering, Beihang University Beijing 100191 China; Key Laboratory of Aerospace Advanced Materials and Performance, Ministry of Education, School of Materials Science and Engineering, Beihang University Beijing 100191 China jyang@buaa.edu.cn

## Abstract

Polyurethanes with different soft–hard segment ratios were successfully synthesized, with an aggregation-induced-emission (AIE)-active tetraaniline derivative (NH_2_–B_3_–Ani_4_–NH_2_) as the hard segment. The resulting polyurethanes exhibited typical AIE features. The fluorescence intensities of polyurethane films changed with heat treatments. The fluorescence intensities of the polyurethane films decreased sharply after quenching treatment, yet their fluorescence intensities exceeded the original intensities of the films after thermal annealing at 80 °C for 24 h. Differential Scanning Calorimetry (DSC) results implied that the melting peaks in polyurethane films disappeared after quenching treatment, but the melting peaks appeared again after thermal annealing. These results proved that the arrangement of the structure had an important effect on the AIE properties of the polyurethane films. Meanwhile, the fluorescence intensities of these polyurethanes decreased with the increase of temperature, indicating that all three polyurethanes exhibited temperature-dependent fluorescent characteristics. Based on the above investigations, the AIE-active polyurethanes may provide a platform for the development of stimuli-responsive fluorescent materials.

## Abstract 2

Polyurethanes with an AIE fluorophore (tetraaniline derivative) are thermo-responsive, demonstrating that AIE-active polyurethane films have promising applications in stimuli-responsive materials.

## Graphical abstract

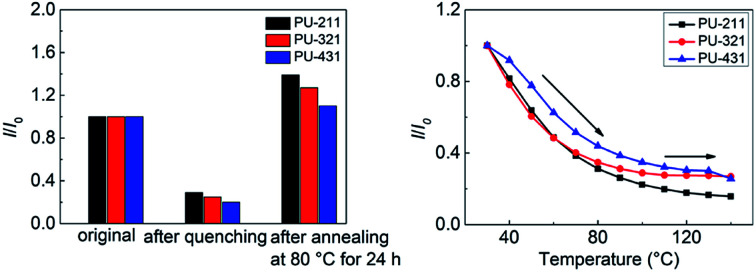

## Introduction

Aggregation-Induced Emission (AIE) has attracted extensive attention since it was first discovered by Tang's group in 2001.^1^ Molecules with AIE characteristics exhibit weak or no emission in solution, but show enhanced emission in the aggregation state or poor solvents. This phenomenon directly offers a straightforward solution to the aggregation-caused quenching (ACQ) problem in the aggregated state.^2^ The mechanism of AIE is ascribed to the restricted intramolecular motion (RIM) in the aggregated state.^3^ Subsequently, a variety of AIE fluorophores have been developed, such as tetraphenylethene,^4^ siloles,^5^ pyrroles,^6^ and distyrylanthracene derivatives.^7^ These fluorophores have been applied in many fields like fluorescence probes, bioimaging and mechano-fluorochromic materials.^8^

Although great progress has been made on small AIE molecules, the investigations of AIE polymers are relatively few.^9^ When small AIE molecules are introduced into polymer chains, the steric hindrance should be imposed between the long chain segments of polymers and small AIE molecules.^10^ In this case, the intramolecular rotations of small AIE molecules are more restricted in polymer systems leading to the higher fluorescent emission, compared to the small AIE molecules.^11^ Furthermore, AIE polymers display more advantages such as high processability, multiple functionalization and simple device fabrication, which are difficult to achieve for small molecule materials.^12^ AIE polymers broaden the application scope of AIE materials and endow them with extensive and promising prospects for practical applications.^13^ To date, a variety of AIE polymers with AIE fluorophore on the side chains or main chains have been successfully designed and synthesized.^14^ However, most researches focus on the introduction of tetraphenylethene derivatives as main fluorophores into polymers to generate the new fluorescent polymers with AIE properties.^15^ Therefore, enriching the family of AIE polymers, and investigating the influence of polymer matrix on the AIE characteristics are beneficial to the development of AIE polymers.

Polyurethane elastomers are composed of two phases, in which the hard segments are arranged orderly and closely to form a crystal zone, contributing to the high strength, rigidity and high melting point; whereas the randomly arrangement of soft segments in amorphous zone gives the elastomer with flexibility, elasticity and low temperature resistance.^16^ When the AIE monomers are imported into the soft segments or the hard segments, the polyurethane will exhibit different AIE performances. Therefore, it is necessary to study the influence of condensed structure on the AIE characteristics.

In our previous research, a new kind of AIE molecule, *N*,*N*′′-bis(4′′-aminophenyl)-*N*,*N*′,*N*′′-tris(2,2-diphenyl vinyl)-4,4′-diaminodiphenylamine (NH_2_–B_3_–Ani_4_–NH_2_), was firstly synthesised with simple method.^17^ In this paper, to enrich the family of AIE polymers, polyurethanes were synthesized by introducing NH_2_–B_3_–Ani_4_–NH_2_ moiety with different ratios of soft/hard segments. These polyurethanes exhibited AIE behaviors. Also, heat treatment could rearrange the condensed structure, leading to great difference in the fluorescence intensity of the AIE polymer.^18^ Based on above description, the three polyurethane films with different ratios of soft/hard segments were processed with a quenching treatment and thermal annealing. The fluorescence intensities of these films decreased sharply after quenching treatment primitively, and finally exceeded those of the original films after thermal annealing. In addition, the fluorescence intensity change became more obvious after annealing treatment with the increase of the hard segment proportion in the polyurethanes. The differential scanning calorimetry (DSC) results proved that the condensed structure of AIE-active polyurethanes had a great influence on the fluorescence intensity. Meanwhile, the fluorescence intensities of these polyurethanes were decreased with the increase of temperature, indicating that the three polyurethanes had temperature-dependent fluorescent behaviors.

## Experimental

### Materials and characterizations

4,4-Diphenylmethane diisocyanate (MDI) and poly(tetrahydrofuran) (*M*_n_ = 1000, PTMG1000) were obtained from Aladdin Chemical Company. 1,4-Butanediol (BDO) and other chemicals reagents were purchased from Beijing Chemical Factory. The PTMG1000 and BDO were dried at 110 °C and 60 °C respectively under vacuum for 2 h before use. *N*,*N*′-Dimethyl formamide (DMF) was distilled after drying fully over calcium hydride (CaH_2_). The other reagents and solvents were used without further purification. ^1^H NMR spectra were completed on a Bruker AV600 spectrometer using deuterated dimethyl sulfoxide (DMSO-d_6_) as solvent at 25 °C. The morphologies of polyurethanes films on ITO glass surface were observed with atomic force microscope (AFM) in tapping mode (Dimension Icon, Veeco). UV-vis absorption spectra were measured on a TU1901 UV-vis spectrometer at room temperature. Fluorescence spectra were obtained using F-7000 fluorescence spectrophotometer. The thermal transitions of the polyurethanes were investigated using a DSC-60 Plus system at a heating rate of 10 °C min^−1^.

### Synthesis of polyurethanes

**Scheme 1:**
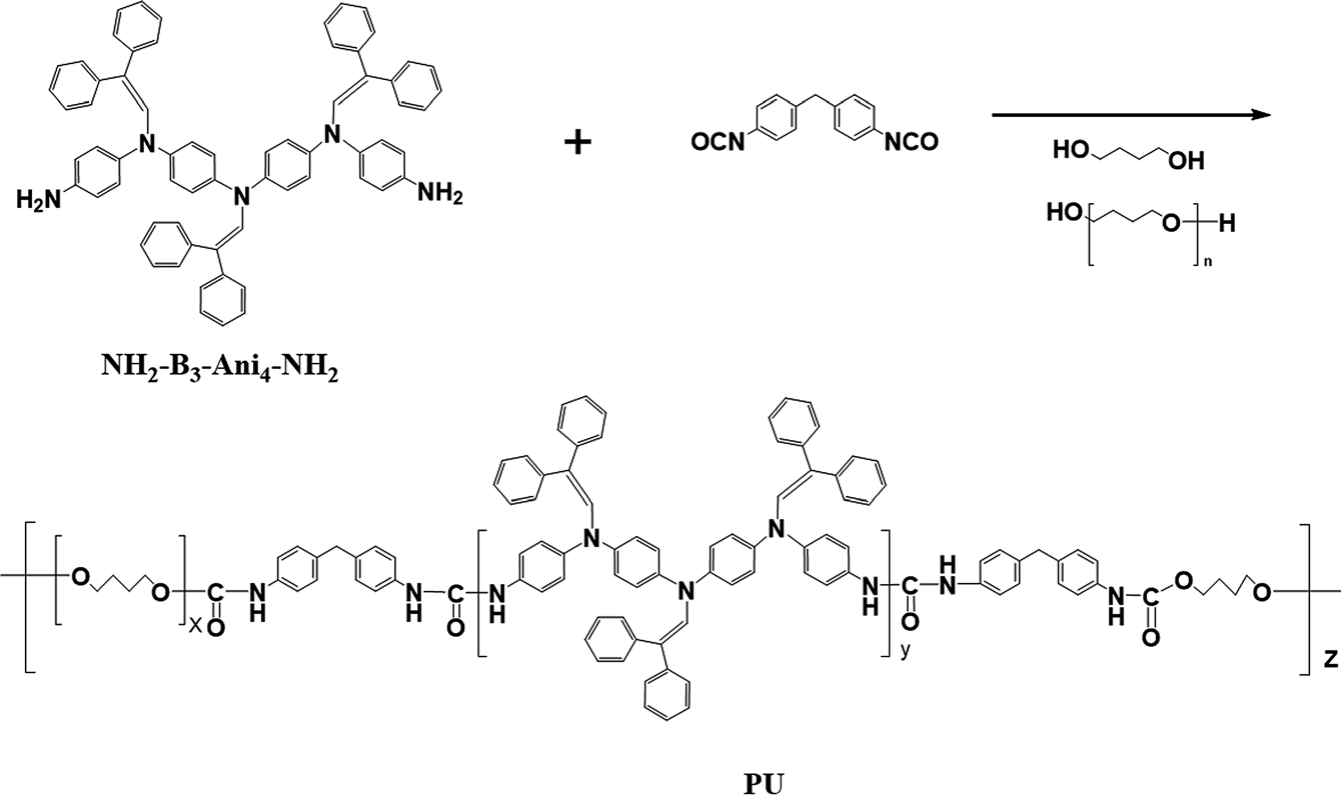
Synthesis rout of the AIE-active polyurethanes.

NH_2_–B_3_–Ani_4_–NH_2_ was synthesized successfully in our previous research.^17^ Detailed experimental procedures were provided in the ESI.† As shown in Scheme 1, MDI and anhydrous DMF were mixed in the flask with magnetic stirring under N_2_ atmosphere. Then PTMG1000 was dissolved in anhydrous DMF and added dropwise into the mixture solution in 0.5 h. After stirring and 0 °C reaction for 1.5 h, a solution of NH_2_–B_3_–Ani_4_–NH_2_ in anhydrous DMF was added into the flask and reacted at 0 °C for 0.5 h. Subsequently, BDO was added dropwise and reacted at room temperature for another 2 h. Finally, the mixture was poured into water, and the resulting precipitates were isolated after being washed with water and ethyl alcohol for several times, then put into vacuum oven until stable weight, yield ∼90%. The reactant ratios for PU-211, PU-321 and PU-431 are listed in Table 1.

**Table 1:** Reactant molar ratios and GPC characterization of three polyurethanes

Polyurethane	Reactant molar ratios	MDI (mmol)	PTMG1000 (mmol)	NH_2_–B_3_–Ani_4_–NH_2_ (mmol)	BDO (mmol)	*M*_n_	*M*_w_	PDI
PU-211	2 : 1 : 1	15.20	7.50	0.066	7.43	43 995	70 239	1.59
PU-321	3 : 2 : 1	15.20	10.00	0.078	4.92	54 809	92 869	1.69
PU-431	4 : 3 : 1	15.20	11.25	0.084	3.67	57 130	96 004	1.68

## Results and discussion

### Synthesis and characterization

As shown in Scheme S1,† the nitro-capped tetraaniline derivative NO_2_–Ani_4_–NO_2_ was firstly synthesized successfully according to the literature.^2^ Then the nitro-capped diphenyl enamine-substituted tetraaniline derivative NO_2_–B_3_–Ani_4_–NO_2_ was synthesized by Schiff Base reaction of secondary amine groups of NO_2_–Ani_4_–NO_2_ and aldehyde groups of diphenylacetaldehyde. Finally, amino-capped tetraaniline derivative NH_2_–B_3_–Ani_4_–NH_2_ was obtained by hydrazine hydrate Pd/C-catalytic reduction of NO_2_–B_3_–Ani_4_–NO_2_. The FTIR, ^1^H-NMR and MALDI MS spectra of NO_2_–Ani_4_–NO_2_, NO_2_–B_3_–Ani_4_–NO_2_ and NH_2_–B_3_–Ani_4_–NH_2_ are presented in Fig. S1–S3.† NH_2_–B_3_–Ani_4_–NH_2_ with AIE characteristic was introduced into the polymers to obtain the AIE-active polyurethanes. PTMG1000 was used as soft segments, then reacted with a mixture of MDI, NH_2_–B_3_–Ani_4_–NH_2_ and BDO with molar ratios of 1 : 2 : 1, 2 : 3 : 1, 3 : 4 : 1, resulting in PU-211, PU-321, PU-431, respectively. The GPC characterization of three polyurethanes is listed in Table 1. Three polyurethanes all have high molecular weight and low polydispersity.

The ^1^H NMR spectrum of PU-211 is presented in Fig. S4.† The chemical shifts 9.64–9.27 ppm were ascribed to the protons of urethane unit (–NH–), while the peak at 8.50 ppm was ascribed to the –NH– protons in NH_2_–B_3_–Ani_4_–NH_2_. The peaks related to the benzene protons appeared at 7.52–6.69 ppm. In addition, the peak related to the –CH_2_O– protons of PTMG1000 appeared at 4.17–3.89 ppm and the peak related to the –CH_2_O– protons of BDO appeared at 3.85–3.67 ppm. A broad and relatively high intensity peak at 2.02–1.00 ppm attributed to –CH_2_ protons of PTMG1000 and BDO. The spectrum proved that the polyurethane was synthesized successfully.

Moreover, the weight fractions of NH_2_–B_3_–Ani_4_–NH_2_ in the AIE polyurethanes were measured by the UV spectrometric method (as shown in Table S1, Fig. S5 and eqn (S1)†) and the results were in accordance with the theoretical results (0.5 wt%), proving that polyurethanes with the NH_2_–B_3_–Ani_4_–NH_2_ moiety in proportions were synthesized successfully.

### AIE behaviors of polyurethanes

**Fig. 1:**
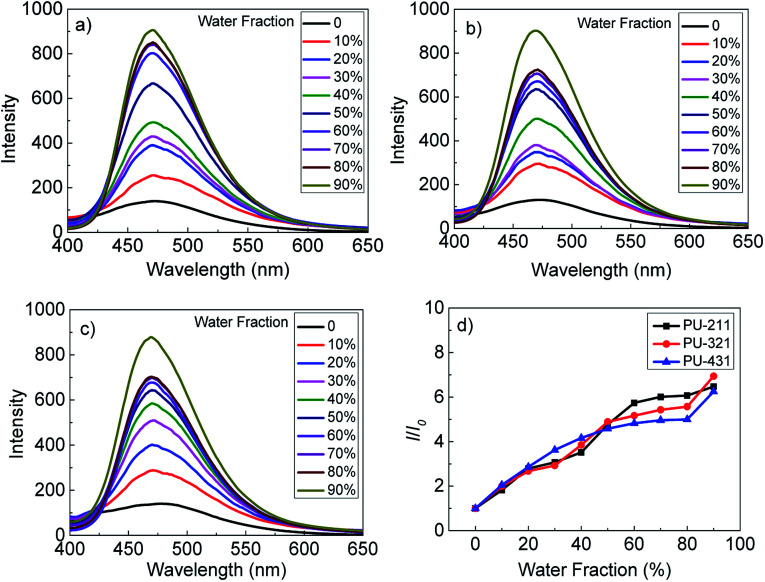
Fluorescence intensity spectra of (a) PU-211, (b) PU-321, (c) PU-431 in DMF/H_2_O mixtures with different water fractions; (d) plots of relative fluorescence intensity (*I*/*I*_0_) of the polyurethanes *versus* the water fraction in DMF/H_2_O solution, *I*_0_: fluorescence intensity of the polyurethanes in pure DMF, mass concentration: 30 mg mL^−1^, excitation wavelength: 350 nm.

The fluorescence emission behaviors of three polyurethanes in DMF/H_2_O mixture with the mass concentration of 30 mg mL^−1^ were investigated. As shown in Fig. 1, The fluorescence intensities of three polyurethanes increased gradually with the addition of water to the DMF solution. When the water fraction reached 90%, the fluorescence intensities increased to the maximum correspondingly. It was found that three polyurethanes had the highest relative fluorescence intensity changes, about 7-fold higher than that in the pure DMF solvent. All the polyurethanes exhibited AIE feature, although the soft and hard segment ratio was different.

To monitor the influence of the water addition on the aggregation state, polyurethane thin films were prepared by coating the sample solution with different water fractions on a glass. AFM was used to investigate the surface morphologies. Taken the PU-211 as an example, as shown in Fig. S6,† the surface were all dot-like structures, and as water addition increased, the dot size increased. The AFM images gave direct evidence that polyurethanes self-assemble into nanoparticles in DMF/H_2_O mixed solution.

### The effect of condensed structure on the fluorescence emission of AIE polyurethanes

**Fig. 2:**
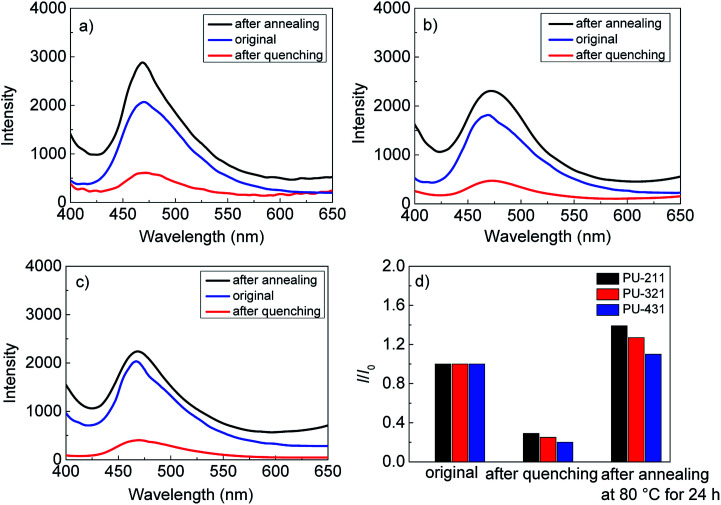
Fluorescence intensity spectra of (a) PU-211, (b) PU-321, (c) PU-431; (d) relative fluorescence intensity changes of the polyurethane films before and after heat treatment.

We systematically researched the influence of the condensed structure on the fluorescence properties. The as-prepared polyurethane films were solution casted-in a PTFE molds and then the films underwent quenching treatments and thermal annealing successively, which are supposed to change the aggregation states of hard AIE segments. Their fluorescence spectra are presented in Fig. 2. The fluorescence intensities of PU-211, PU-321 and PU-431 films decreased sharply after quenching treatment, however, exceeding those of the original films after thermal annealing at 80 °C for 24 h. On the other hand, the changes of the fluorescence intensity were less obvious after annealing treatment with the decrease of the hard segment ratio from 75% to 62.5% in the polyurethanes.

**Fig. 3:**
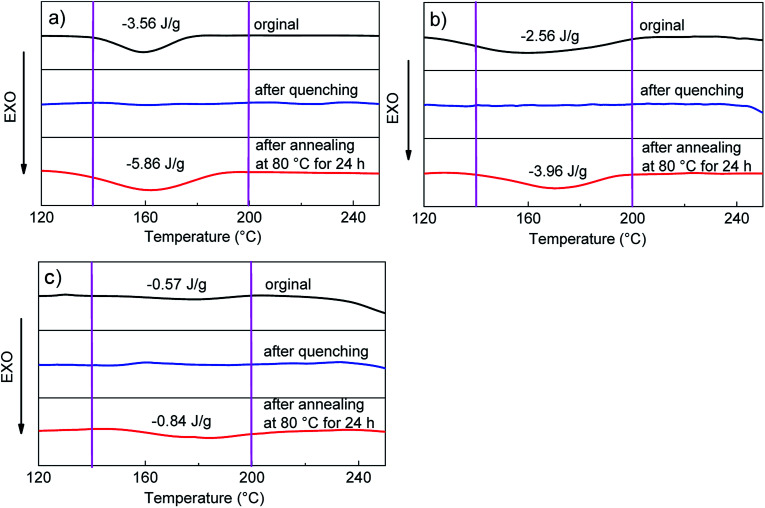
DSC traces of (a) PU-211, (b) PU-321 and (c) PU-431 before and after the heat treatment.

The effect of aggregation state structure on fluorescence properties of the polyurethanes was further investigated by DSC. As shown in Fig. 3, there was a broad peak in the range of 140–200 °C in the original films, which corresponds to the melting point of the hard segments in the polyurethanes. The endothermic peaks disappeared after quenching treatment, implying that the polyurethanes after quenching treatment had an irregular hard segment structure and resulted in the decrease of fluorescence intensity. Then the melting peaks appeared again and became more apparent after heat treatment at 80 °C for 24 h, which indicated that thermal annealing treatment could effectively rearrange the hard segments to form a more regular structure. Compared with the original state, the thermal enthalpy of melting peaks decreased with the increase of the hard segments before and after thermal annealing treatment, which corresponds to the changes of fluorescence intensity of PU-211, PU-321 and PU-431 in Fig. 2d. PU-211 had a larger ratio of regular structure after the annealing treatment, leading to the restriction of intramolecular rotation of the diphenyl amine rotors in the hard segments and consequently the large enhancement of the fluorescence intensity.

The melting points of the hard segments of the three polyurethanes was 140–200 °C in the original films (Fig. 3). At low temperature, NH_2_–B_3_–Ani_4_–NH_2_ units in hard segments of polyurethanes were in partly crystals state. Hence the intramolecular motion of diphenyl amine of NH_2_–B_3_–Ani_4_–NH_2_ were restricted, which enabled excitons to decay radiative and thus emitted efficiently at low temperatures.^19^ At high temperature, however, polyurethane crystals melted, and thus intramolecular motions of NH_2_–B_3_–Ani_4_–NH_2_ were activated, which consumed the energy of excitons through nonradiative relaxations, leading to a weak emission.

In summary, the condensed structure of the polymer matrix had a positive effect on the fluorescence intensity of the AIE polymers. Meanwhile, the organized structure of the polymer matrix can improve the fluorescence emissions of AIE polymers and promote their application in fluorescent probes, stimuli-responsive materials, PLED devices and so on.^16^

### The effect of temperature on the fluorescence emission of AIE polyurethanes

**Fig. 4:**
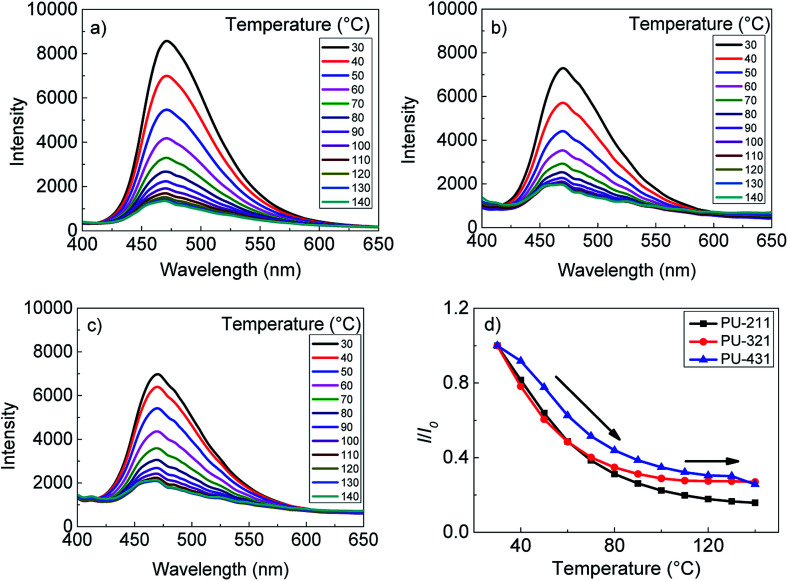
Temperature-dependent emission spectra of (a) PU-211, (b) PU-321 and (c) PU-431 films from 30 to 140 °C; (d) fluorescence intensities of PU-211, PU-321 and PU-431 *versus* the temperature.

The fluorescence intensity of polyurethanes was affected by the structure of polymer, which could be tuned by temperature. The relationship between the fluorescence intensity of the AIE polyurethanes films and temperature was investigated. The fluorescence spectra of PU-211, PU-321 and PU-431 at various temperatures from 30 °C to 140 °C were shown in Fig. 4a–c. All three polyurethanes had temperature-dependent fluorescence behaviors. The three polyurethane films emitted strong fluorescence at 30 °C. Afterwards, the fluorescence intensity decreased gradually with the increasing temperature. The fluorescence intensities of PU-211, PU-321 and PU-431 were gradually decreased by about 85%, 73% and 74% respectively (seen in Fig. 4d). The variation of fluorescence intensity investigated for PU-211 was much more obvious than that of PU-321 and PU-431.

It was noted that the fluorescence intensities of three polyurethanes decreased more drastically at low temperature, indicating that the polyurethane films were more sensitive to thermal expansion below *T*_m_ than to segmental movements near *T*_m_. The possible reason was that DSC detects heat variation corresponding to the evolution of polymer crystals during crystallization or melting process, while fluorescence intensity was affected by a few temperature-related factors including polymer crystals, viscosity and so on.^20^

## Conclusions

In summary, three polyurethanes were successfully designed and synthesized with different ratios of soft/hard segments. The mixture of MDI and NH_2_–B_3_–Ani_4_–NH_2_ as hard segments was reacted with PTMG1000 and BDO in the molar ratio of 2 : 1 : 1, 3 : 2 : 1 and 4 : 3 : 1 to give desired polyurethanes named PU-211, PU-321 and PU-431, respectively. Their fluorescence emission intensities gradually increased with increased water fraction in DMF, which exhibited typical AIE phenomenon. These three polyurethane films showed remarkable thermal treatment effect on the fluorescence emission: the fluorescence intensities of these polyurethane films decreased drastically after quenching and increased even higher than the original subsequently thermal annealing. Interestingly, the change of fluorescence intensity became more obvious after annealing treatment with the increase of the hard segments in the polyurethanes. DSC characterizations revealed that thermal treatment caused more regular arrangement of condensed structure, resulting in higher fluorescence intensity. Moreover, the polyurethane films with AIE fluorophore had the temperature-dependent emission behaviors. In brief, the structure of the polymer, such as the soft and hard segment ratio and condensed state, played an important factor in the fluorescence emission of the AIE polymers.

## Conflicts of interest

There are no conflicts to declare.

## Supplementary Material

RA-010-D0RA06193J-s001

## References

[cit1] Luo J., Xie Z., Lam J. W. Y., Cheng L., Chen H., Qiu C., Kwok H. S., Zhan X., Liu Y., Zhu D., Tang B. Z. (2001). Chem. Commun..

[cit2] Liu B., Wang K., Lu H., Huang M., Yang J. (2019). New J. Chem..

[cit3] Chen J., Law C. C. W., Lam J. W. Y., Dong Y., Lo S. M. F., Williams I. D., Zhu D., Tang B. Z. (2003). Chem. Mater..

[cit4] Zhang Y., Mao H., Xu W., Shi J., Cai Z., Tong B., Dong Y. (2018). Chem.–Eur. J..

[cit5] Li Z., Dong Y. Q., Lam J. W. Y., Sun J., Qin A., Häußler M., Dong Y. P., Sung H. H. Y., Williams I. D., Kwok H. S., Tang B. Z. (2009). Adv. Funct. Mater..

[cit6] Shi X., Wang H., Han T., Feng X., Tong B., Shi J., Zhi J., Dong Y. (2012). J. Mater. Chem..

[cit7] Chen M., Chen R., Shi Y., Wang J., Cheng Y., Li Y., Gao X., Yan Y., Sun J. Z., Qin A., Kwok R. T. K., Lam J. W. Y., Tang B. Z. (2018). Adv. Funct. Mater..

[cit8] Kanekar D. N. N., Chacko S., Kamble R. M. M. (2020). New J. Chem..

[cit9] Huang Z., Wang R., Chen Y., Liu X., Wang K., Mao L., Wang K., Yuan J., Zhang X., Tao L., Wei Y. (2019). Polym. Chem..

[cit10] Wang K., Lu H., Liu B., Yang J. (2018). Tetrahedron Lett..

[cit11] Hu Y., Lam J. W. Y., Tang B. Z. (2019). Chin. J. Polym. Sci..

[cit12] Liu J. Z., Lam J. W. Y., Tang B. Z. (2009). Chem. Rev..

[cit13] Yang Y. M., Zhao Q., Feng W., Li F. Y. (2013). Chem. Rev..

[cit14] Liu X., Chen T., Yu F., Shang Y., Meng X., Chen Z. R. (2020). Macromolecules.

[cit15] Dong Y., Lam J. W. Y., Qin A., Liu J., Li Z., Tang B. Z., Sun J., Kwok H. S. (2007). Appl. Phys. Lett..

[cit16] Wang K., Lu H., Liu B., Yang J. (2018). Eur. Polym. J..

[cit17] Liu B., He W., Lu H., Wang K., Huang M., Kwok R. T. K., Lam J. W. Y., Gao L., yang J., Tang B. Z. (2019). Sci. China: Chem..

[cit18] Wang K., Yang J., Gong C., Lu H. (2017). Faraday Discuss..

[cit19] Liu C. L., Lin M. C., Chen H. L., Műller A. J. (2015). Macromolecules.

[cit20] Wu J. L., Zhang C., Qin W., Quan D. P., Ge M. L., Liang G. D. (2019). Chin. J. Polym. Sci..

