# Supplementary material for: Thermally responsive AIE-active polyurethanes based on a tetraaniline derivative
Source: RSC Adv. 2020 Nov 13;10(68):41424–9. doi: 10.1039/d0ra06193j (PMC9057807; doi:10.1039/d0ra06193j)
Supplement: RA-010-D0RA06193J-s001 [file RA-010-D0RA06193J-s001.pdf]

Electronic Supplementary Material (ESI) for RSC Advances.
This journal is © The Royal Society of Chemistry 2020

Electronic Supplementary Material (ESI) for RSC Advances
This journal is © The Royal Society of Chemistry 2020

Supporting Information

Thermally responsive AIE-active polyurethanes based on a tetraaniline derivative

Beibei Liu,[a] Kun Wang,[b] Hao Lu,[b] Mingming Huang,[b] Zhigang Shen[a] and Jiping Yang*[b]

a: Beijing Key Laboratory for Powder Technology Research & Development, School of Aeronautical Science and Engineering, Beihang University, Beijing, 100191, China
b: Key Laboratory of Aerospace Advanced Materials and Performance, Ministry of Education, School of Materials Science and Engineering, Beihang University, Beijing 100191, China
E-mail: jyang@buaa.edu.cn

1. Synthesis

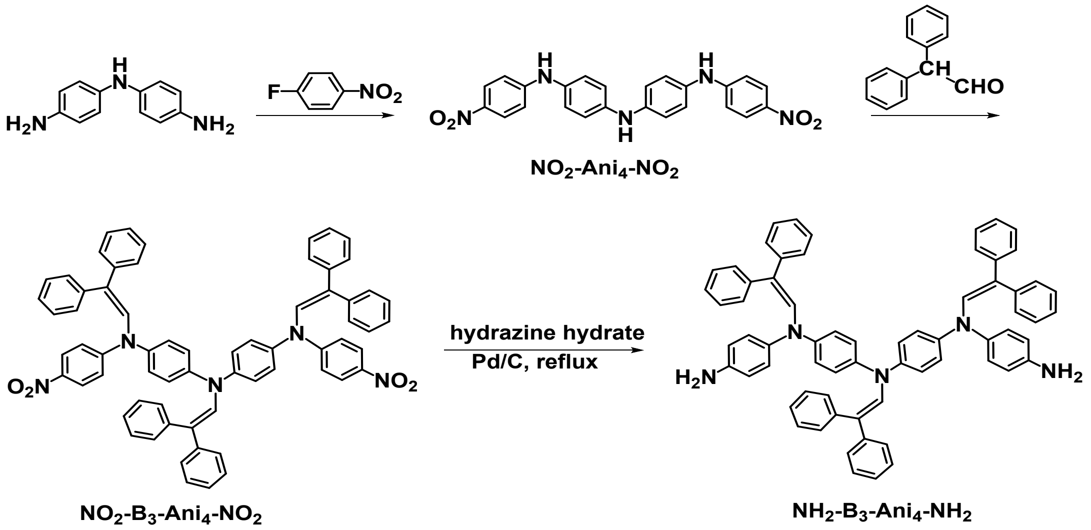

Scheme S1 Synthetic route of NH_2-B_3-Ani_4-NH_2.

1.1 N,N-bis(4''-nitrophenyl)-4,4''-diaminodiphenylamine (NO_2-Ani_4-NO_2)

4,4'-Diaminodiphenylamine sulfate hydrate (9.22 g, 31 mmol) and triethylamine (9.95 g, 98 mmol) were dissolved in 50 mL of dimethyl sulfoxide (DMSO) at room temperature, and 4-fluoro-nitrobenzene (11.03 g, 78 mmol) was added in sequence. The mixture was stirred at 90 °C for 72 h under nitrogen atmosphere to avoid amine oxidation. After the mixture was cooled to room temperature and poured into stirred water, the resulting precipitates were collected and purified by reprecipitation from tetrahydrofuran (THF) to give desired **NO_2-Ani_4-**

NO_2 as brown powder (11.62 g, yield 85%). FTIR (KBr, cm^{-1}): 3356 (N-H); 2975, 2913, 2848 (C-H, Ar); 1600, 1297 (NO_2); 1113 (C-N); 831, 750, 694 (C-H, Ar). ^1H NMR (600 MHz, DMSO-d_6), δ, ppm: 9.12 (s, 2H, C-NH), 8.22 (s, H, C-NH), 8.04 (d, 4H, Ar-H), 7.16-7.10 (m, 8H, Ar-H), 6.90 (d, 4H, Ar-H). MS (MALDI-TOF): m/z: 440.9 (M^+ calcd 441.1).

1.2 N,N''-bis[(4''-nitrophenyl)-N,N',N''-tris(2,2-diphenyl-vinyl)-4,4'-diaminodiphenylamine (NO_2-B_3-Ani_4-NO_2)

NO_2-Ani_4-NO_2 (0.882 g, 2 mmol), camphorsulfonic acid (0.02 g, 0.08 mmol) and diphenyl acetaldehyde (1.177 g, 6 mmol) were dissolved in THF (30 mL), and the resulting mixture was heated to reflux for 24 h under nitrogen atmosphere with stirring. Then the solvent was removed by rotary evaporation, and the left crude product was purified by recrystallization from ethyl acetate (EA) to give **NO_2-B_3-Ani_4-NO_2** as red powder (1.70 g, yield 87%). FTIR (KBr, cm^{-1}): 3058, 2920, 2853 (C-H, Ar); 1586, 1311 (NO_2); 1502 (C=C); 1113 (C-N); 840, 750, 699 (C-H, Ar). ^1H NMR (600 MHz, DMSO-d_6), δ, ppm: 8.08 (d, 4H, Ar-H), 7.13-7.34 (m, 30H, Ar-H), 6.87 (d, 8H, Ar-H), 6.78 (d, 4H, Ar-H), 6.55-6.60 (m, 3H, CH=C). MS (MALDI-TOF): m/z: 975.3 (M^+ calcd 975.3).

1.3 N,N''-bis(4''-aminophenyl)-N,N',N''-tris(2,2-diphenylvinyl)-4,4'-diaminodiphenylamine (NH_2-B_3-Ani_4-NH_2)

NO_2-B_3-Ani_4-NO_2 (1.0 g, 1.02 mmol) and 10% Pd/C (0.1 g) were dispersed into ethyl alcohol, and added into the three-neck flask with magnetic stirring under nitrogen atmosphere. Then 10 mL hydrazine hydrate was added dropwise into the flask. After it was stirred and refluxed for 24 h, the mixture was filtered to remove Pd/C, then the filtrate was collected and cooled to room temperature to separate out the product **NH_2-B_3-Ani_4-NH_2** as yellow powder after dried in vacuo at 50 °C (0.83 g, yield 89%). FTIR (KBr, cm^{-1}): 3291, 3045 (NH_2); 1500 (C=C); 1256 (C-N); 830, 764, 697 (C-H, Ar). ^1H NMR (600 MHz, DMSO-d6), δ, ppm: 7.30-7.00 (m, 30H, Ar-H), 6.85 (s, 3H, CH=C), 6.57-6.40 (m, 16H), 4.84 (s, 4H, NH_2). MS (MALDI-TOF): m/z: 915.3 (M+ calcd 915.4).

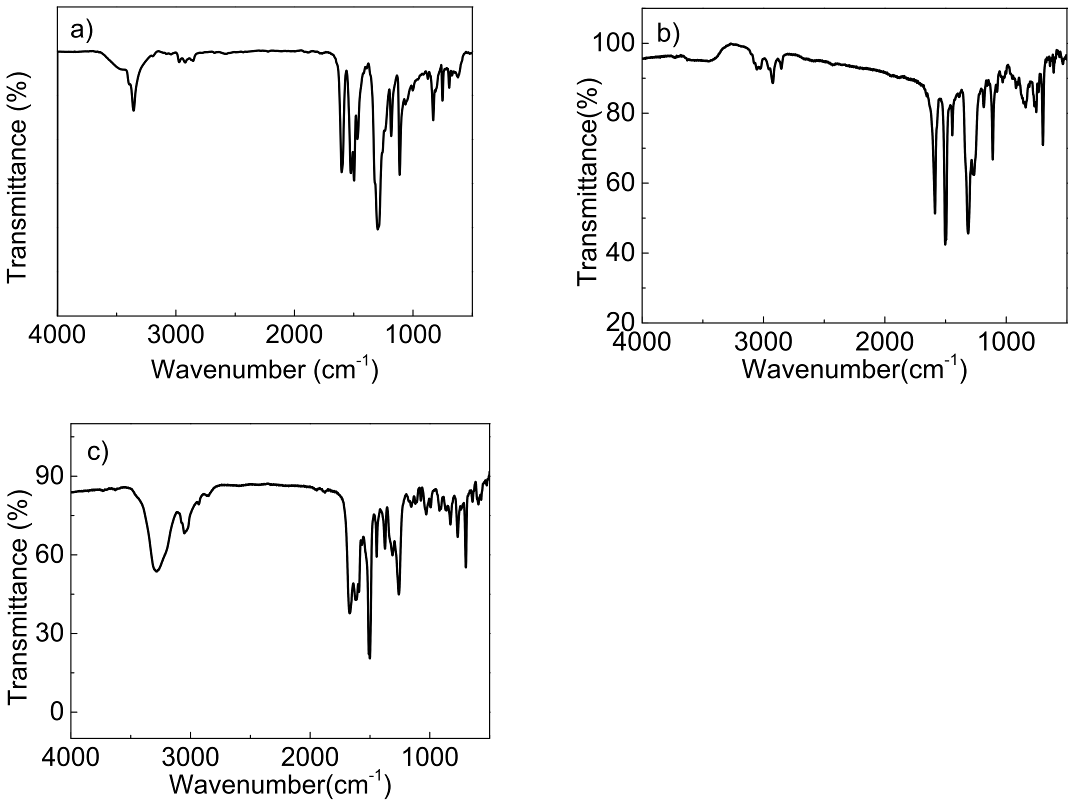

Fig. S1 FTIR spectra of a) NO_2- Ani_4-NO_2; b) NO_2-B_3-Ani_4-NO_2; c) NH_2-B_3-Ani_4-NH_2.

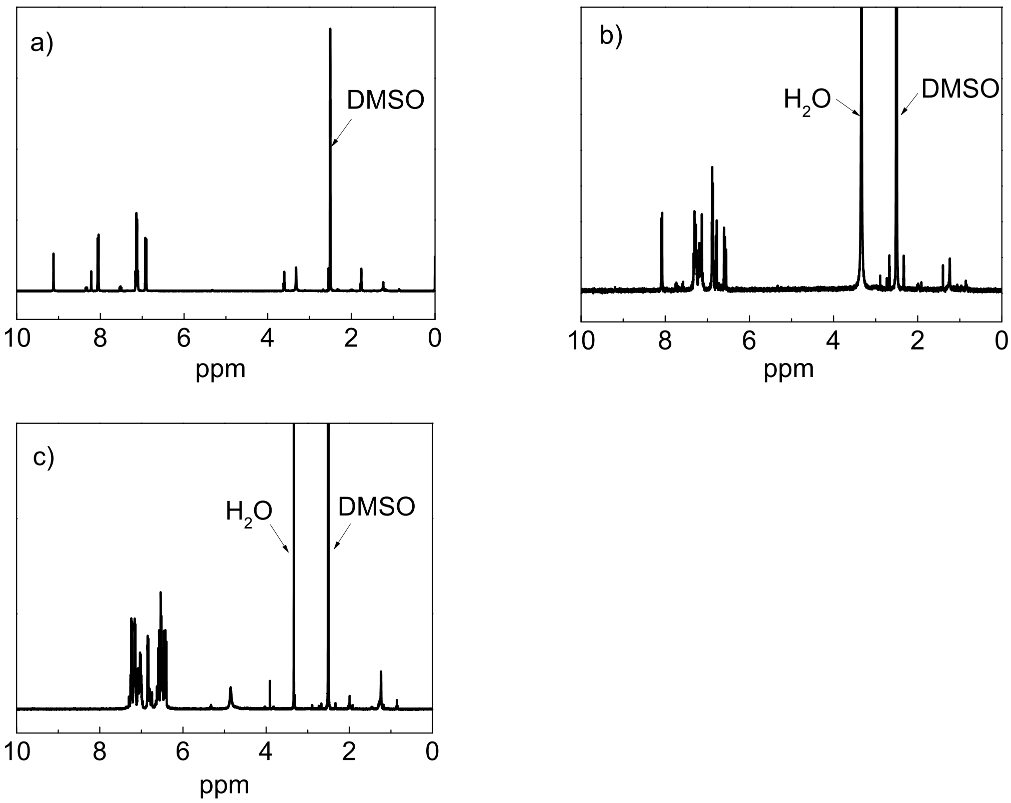

Fig. S2 ^{1}H-NMR spectra of a) NO_2- Ani_4-NO_2; b) NO_2-B_3-Ani_4-NO_2; c) NH_2-B_3-Ani_4-NH_2.

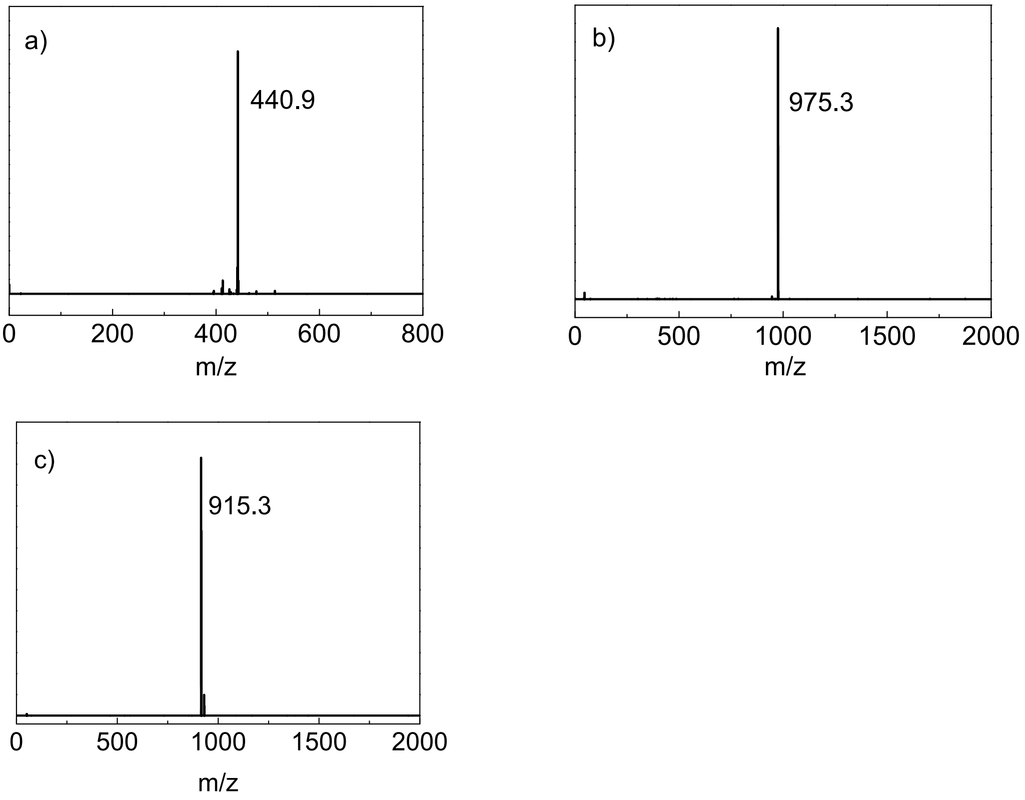

Fig. S3 MALDI MS spectra of a) NO_2-Ani_4-NO_2; b) NO_2-B_3-Ani_4-NO_2; c) NH_2-B_3-Ani_4-NH_2.

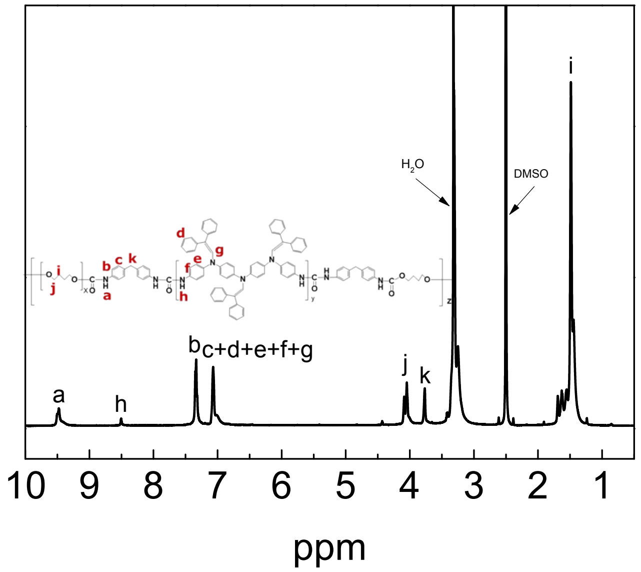

Fig. S4 ^{1}H-NMR spectrum of PU-211.

2. UV spectrometric method

Standard NH_2-B_3-Ani_4-NH_2 solutions with concentrations ranged from 0.001 mg/mL to 0.02 mg/mL were prepared by volumetric flasks. Then the UV spectra of NH_2-B_3-Ani_4-NH_2 solutions were showed in Fig. S5 a). Since the peak at 350 nm was ascribed to the characterized benzene group of NH_2-B_3-Ani_4-NH_2, it was selected to be a standard peak to

measure the mass concentrations of NH_2-B_3-Ani_4-NH_2 in three polyurethanes. As described in Figure S5 b), the absorption peak intensity at 350 nm was in proportion to the mass concentration of NH_2-B_3-Ani_4-NH_2. Then the Equation S1 could be obtained by fitting the above experimental data, in which *c* was the NH_2-B_3-Ani_4-NH_2 mass concentration in polyurethane solution and *A* was the UV absorbance of the corresponding polyurethane solution. By measuring the UV absorbances of three polyurethane solutions which have a same solution concentration of 3.0 mg/mL, NH_2-B_3-Ani_4-NH_2 mass concentrations of three polyurethanes could be calculated with Equation S1.

$$A=56.63721C + 0.01084\ (R^2=0.9994) \qquad \text{Equation S1}$$

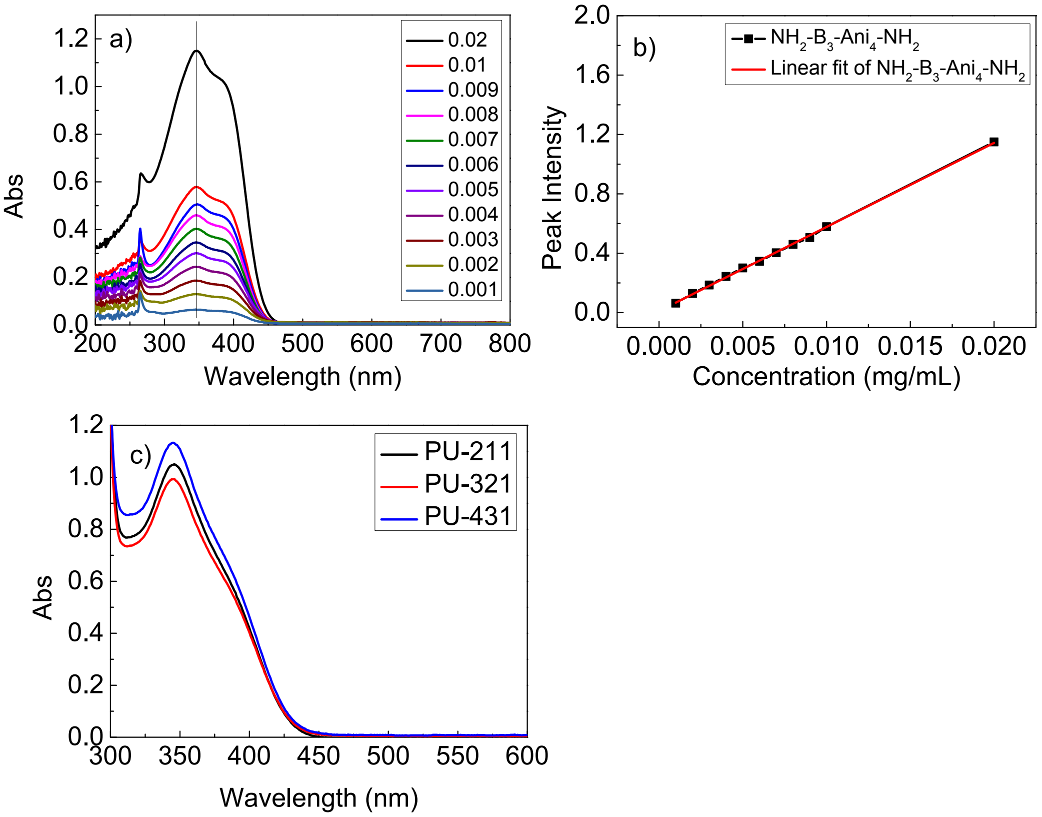

Fig. S5 a) UV spectra of NH_2-B_3-Ani_4-NH_2 at different concentration; b) Plot of UV peak intensity and NH_2-B_3-Ani_4-NH_2 solution concentration; c) UV-vis absorption spectra of three polyurethanes, concentration: 3.0 mg/mL.

Table S1. Mass fraction of NH_2-B_3-Ani_4-NH_2 in the three modified polyurethanes.

Polyurethane	UV absorbance	Experimental mass fraction (wt%)	Theoretical mass fraction (wt%)
**PU-211**	1.05	0.61	0.51
**PU-321**	0.99	0.58	0.51
**PU-431**	1.13	0.66	0.51

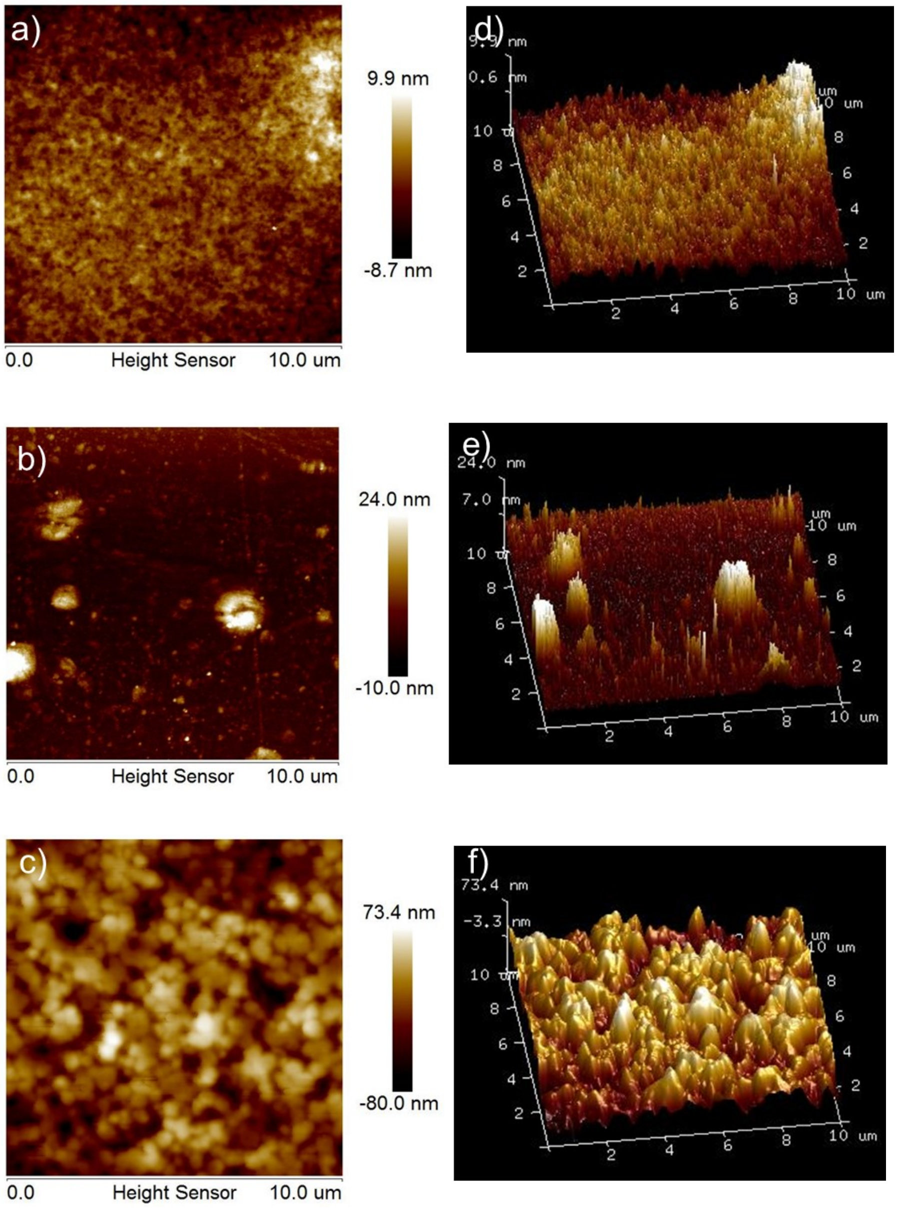

Fig. S6 AFM images of PU-211 thin films prepared by mixed solutions of different water fractions: (a), (b) and (c) are high contrast images at water fraction=0%, 40% and 80%, respectively; (d), (e) and (f) are the corresponding 3D high contrast images of (a), (b) and (c), respectively.
